# Lipoxin A4 (LXA4) as a Potential Drug for Diabetic Retinopathy

**DOI:** 10.3390/medicina61020177

**Published:** 2025-01-21

**Authors:** Undurti N. Das

**Affiliations:** UND Life Sciences, 2221 NW 5th St, Battle Ground, WA 98604, USA; undurti@hotmail.com; Tel.: +1-508-904-5376

**Keywords:** diabetic retinopathy, AMD, inflammation, lipoxin A4, cytokines, SARS-CoV-2, angiogenesis

## Abstract

The purpose of this review is to propose that lipoxin A4 (LXA4), derived from arachidonic acid (AA), a potent anti-inflammatory, cytoprotective, and wound healing agent, may be useful to prevent and manage diabetic retinopathy (DR). LXA4 suppresses inappropriate angiogenesis and the production of pro-inflammatory prostaglandin E2 (PGE2), leukotrienes (LTs), 12-HETE (12-hydroxyeicosatetraenoic acid), derived from AA by the action of 12-lioxygenase (12-LOX)) interleukin-6 (IL-6), and tumor necrosis factor-α (TNF-α), as well as the expression of NF-κB, inducible NO (nitric oxide) synthase (iNOS), cyclooxygenase-2 (COX-2), intracellular adhesion molecule-1 (ICAM-1), and vascular endothelial growth factor (VEGF)—factors that play a role in DR. Thus, the intravitreal injection of LXA4 may form a new approach to the treatment of DR and other similar conditions such as AMD (age-associated macular degeneration) and SARS-CoV-2-associated hyperinflammatory immune response in the retina. The data for this review are derived from our previous work conducted in individuals with DR and from various publications on LXA4, inflammation, and DR.

## 1. Introduction

Diabetic retinopathy (DR) is the leading cause of blindness among adults aged 20 to 74 years who have diabetes mellitus (DM) [1]. Vision loss due to DR may occur due to retinal detachment, vitreous hemorrhage, associated neovascular glaucoma, and macular edema or capillary nonperfusion [2]. The presence of DR suggests that microcirculatory dysfunction may be present in other organs as well [3,4]. DR costs the United States approximately USD 500 million annually [5]. Vision loss due to DR can be reduced by the effective control of diabetes and blood pressure and by early detection and treatment [6,7,8].

### 1.1. DR Is a Pro-Inflammatory Condition

Increased vascular permeability; the breakdown of the blood–retinal barrier (BRB); the apoptotic death of retinal neurons, endothelial cells (ECs), and pericytes; and the appearance of microaneurysms occur in DR. In early DR, the degeneration of retinal capillaries occurs, which renders the retina ischemic, resulting in retinal neovascularization. The pro-inflammatory events in DR include (i) enhanced pro-inflammatory cytokine production; (ii) leukostasis; (iii) increased vascular permeability; and (iv) the activation of nuclear factor-κB (NF-κB); enhanced expression of inducible NO (nitric oxide) synthase (iNOS), cyclooxygenase-2 (COX-2), and intracellular adhesion molecule-1 (ICAM-1); and excess production of vascular endothelial growth factor (VEGF) [9,10,11,12].

Placental growth factor (PlGF), a member of the VEGF family expressed by endothelial cells (ECs) and retinal pigment epithelial cells (RPEs) [13,14], also plays a role in DR. PlGF binds to fms-like tyrosine kinase-1 (FLT1; also known as VEGFR-1) and soluble FLT1, a circulating form of FLT1. The activation of FLT1 by PlGF enhances the effects of VEGF signaling, suggesting synergistic effects of PlGF and VEGF [15]. PlGF forms heterodimers with VEGF [8] and exerts pro-angiogenic effects on ECs [13]. In DR, retinas show a higher expression of PlGF [16], which may be localized to endothelial and perivascular regions of neovascular membranes [17]. PlGF is produced by human RPE cells in response to hypoxia [18]. PlGF levels are higher in aqueous and vitreous humor in DR [19,20,21]. PlGF protein expression is upregulated in the vascular endothelial cells of individuals with diabetes. PIGF deficiency reverses the increased expression of tight junction protein ZO-1 and VE-cadherin in diabetes. PlGF deletion enhances Akt phosphorylation. In diabetes, the hypoxia-inducible factor (HIF)1α–VEGF pathway is activated. The increased expression of HIF1α, VEGF, VEGFR1–3, phospho (p)-VEGFR1, p-VEGFR2, and p-endothelial nitric oxide synthase that is seen in DR is inhibited in the retinas of diabetic PlGF^−/−^ mice, with no changes in the enhanced expression of intercellular adhesion molecule-1 (ICAM-1), vascular cell adhesion molecule-1 (VCAM-1), CD11b, or CD18. These results suggest that although PlGF is needed for the development of DR, its genetic deletion protects the retina from diabetic damage. Thus, PlGF alone is not sufficient to produce all the features of DR. The prevention of DR seems to require Akt activation and HIF1α–VEGF pathway inhibition [22].

These results imply that the suppression of the excess production of pro-inflammatory cytokines, reduction in vascular permeability by stabilizing vascular endothelial cells and pericytes [23], and inhibition of expression of nuclear factor-κB (NF-κB), iNOS, COX-2, ICAM-1, and VEGF (including PlGF) are essential to prevent DR.

### 1.2. Bioactive Lipids in the Retina

The retina, especially the retinal pigment epithelium (RPE) is rich in docosahexaenoic acid (DHA, 22:6 n-3) and arachidonic acid (20:4 n-6) (DHA > AA). [24,25]. DHA and AA are needed for RPE function and to prevent retinal degeneration due to oxidative stress [26]. DHA augments the RPE generation of pigment epithelial-derived factor (PEDF), which exhibits cytoprotective actions, especially against oxidative stress [27]. Pericytes that are in close contact with endothelial cells are needed for the maintenance of the blood–retinal barrier. In diabetes, this close interaction between pericytes and endothelial cells is lost. Hyperglycemia enhances protein kinase C- δ (PKC-δ) expression. This blocks platelet derived growth factor (PDGF) signaling to Akt (a serine/threonine kinase), which leads to pericyte cell death. The loss of pericytes, increase in VEGF expression, activation of PKC-β in endothelial cells, loss of the junctional complex, and increase in vascular permeability are all factors that contribute to vascular angiogenesis and DR [28].

### 1.3. Eicosanoids in DR

**Figure 1:**
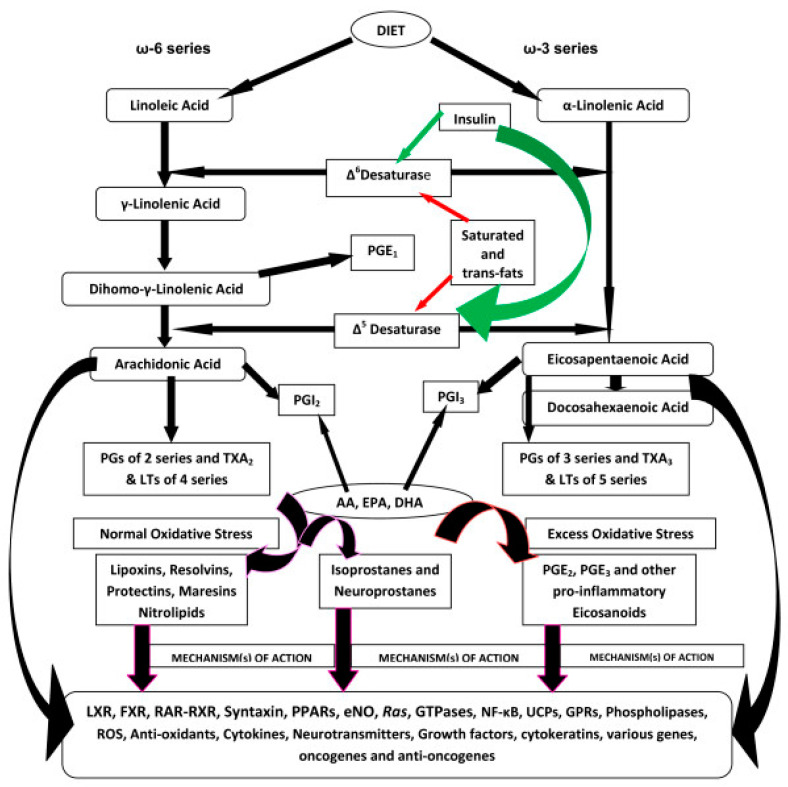
Scheme showing the metabolism of essential fatty acids (EFAs) and various metabolites formed from them, as well as their potential actions regarding inflammation.

**Figure 2:**
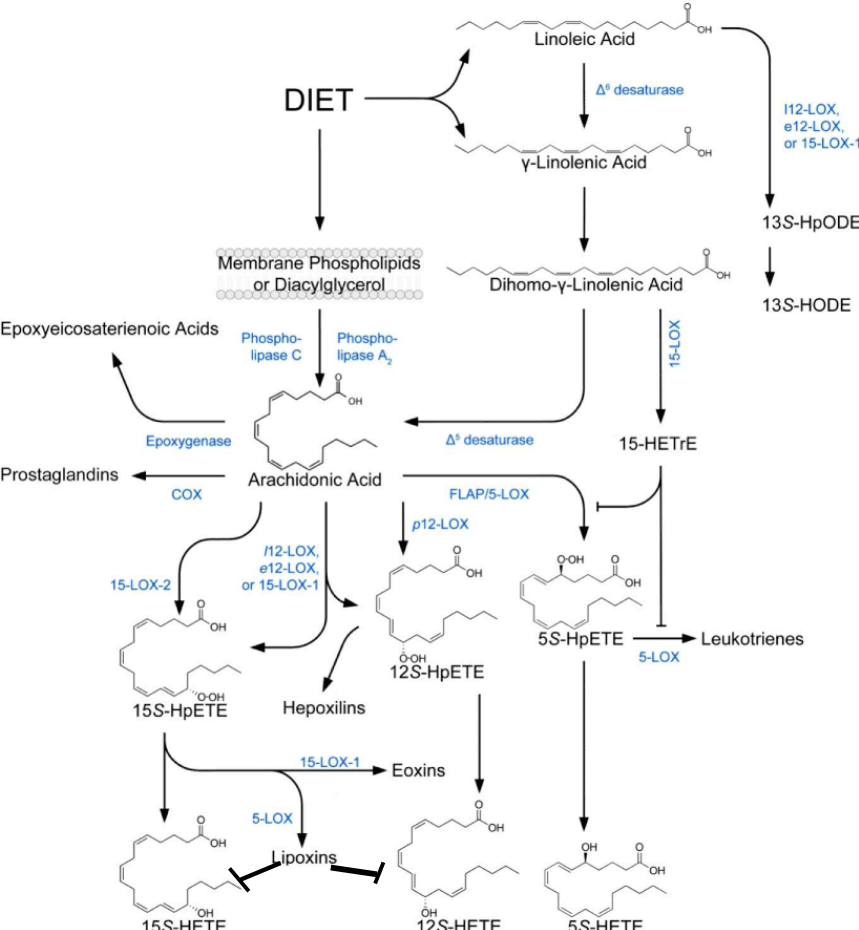
Scheme showing the metabolism of AA from dietary LA. The potential balance between HETEs and LXA4 is also depicted.

**Figure 3:**
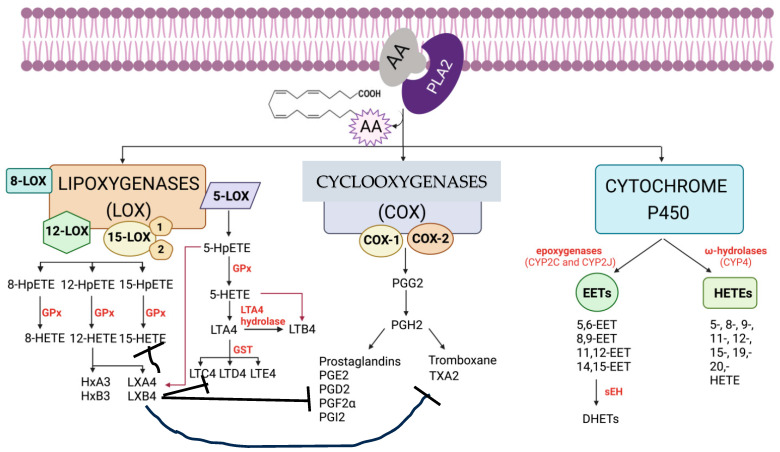
Scheme showing the metabolism of AA by COX, LOX, and cytochrome P450 enzymes.

**Figure 4:**
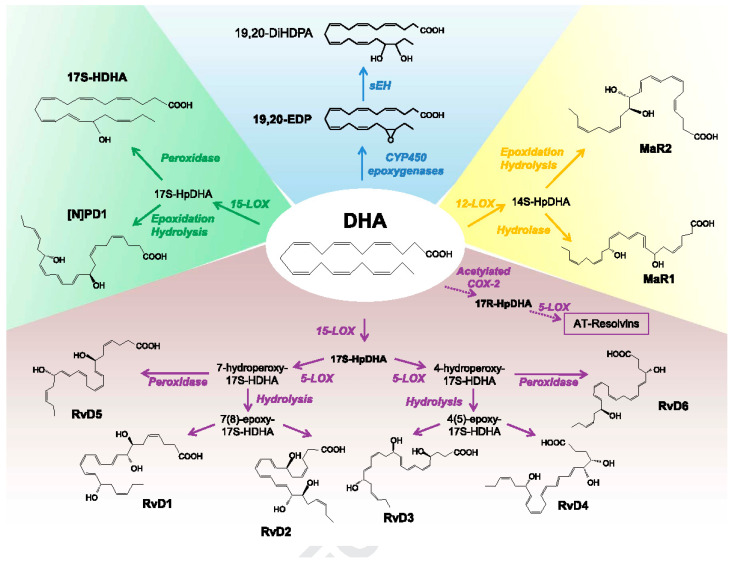
Lipoxygenase products of DHA—resolvins of D series (RvDs), protectins (PDs), and maresins (MaRs)—that have anti-inflammatory actions.

The enhanced production of pro-inflammatory metabolites of AA {such as prostaglandin E2 (PGE2) and leukotrienes (LTs)} and decreased production of anti-inflammatory (from AA and DHA) lipoxin A4 (LXA4), resolvins, protectins, and maresins occurs in DR [29,30,31,32,33,34,35,36,37,38]. This implies that restoring this imbalance between pro- and anti-inflammatory eicosanoids may be of benefit in DR. Under normal physiological conditions, PGE2 and LT synthesis is suppressed by LXA4, resolvins, protectins, and maresins (see Figure 1, Figure 2, Figure 3 and Figure 4). Hence, one method of restoring an imbalance that is tilted more toward PGE2 and LTs could be the local injection/administration of LXA4, resolvins, protectins, and maresins [39,40,41,42,43].

Wild-type and 12/15-lipoxygenase-deficient but not 5-lipoxygenase-deficient mice develop the degeneration of retinal capillaries and pericytes and show an increase in leukostasis and enhanced superoxide production. This indicates that pro-inflammatory metabolites of 5-lipoxygenase play a role in DR [29]. In contrast, the 5-lipoxygenase oxidation product of DHA, 4-hydroxy-docosahexaenoic acid (4-HDHA), the precursor of resolvin D3 and D4 (which have anti-inflammatory and antioxidant actions) inhibited endothelial cell proliferation and angiogenesis via peroxisome proliferator-activated receptor γ (PPARγ) ([44] and see Figure 1, Figure 2, Figure 3 and Figure 4). This implies that 4-HDHA and resolvins are of significant benefit in the prevention and management of DR.

A recent study showed that neuroprotectin D1 (NPD1, derived from DHA) counteracts the H2O2/tumor necrosis factor-α/oxidative stress-triggered apoptosis of RPE cells [44,45]. We reported that the plasma and vitreous content of brain-derived neurotrophic factor (BDNF) and the anti-inflammatory metabolite of AA, lipoxin A4, are low, with a concomitant increase in IL-6 and VEGF and no change in PEDF in individuals with DR ([46], see Table 1, Table 2, Table 3 and Table 4).

### 1.4. Altered EFA Metabolism in DR

**Figure 5:**
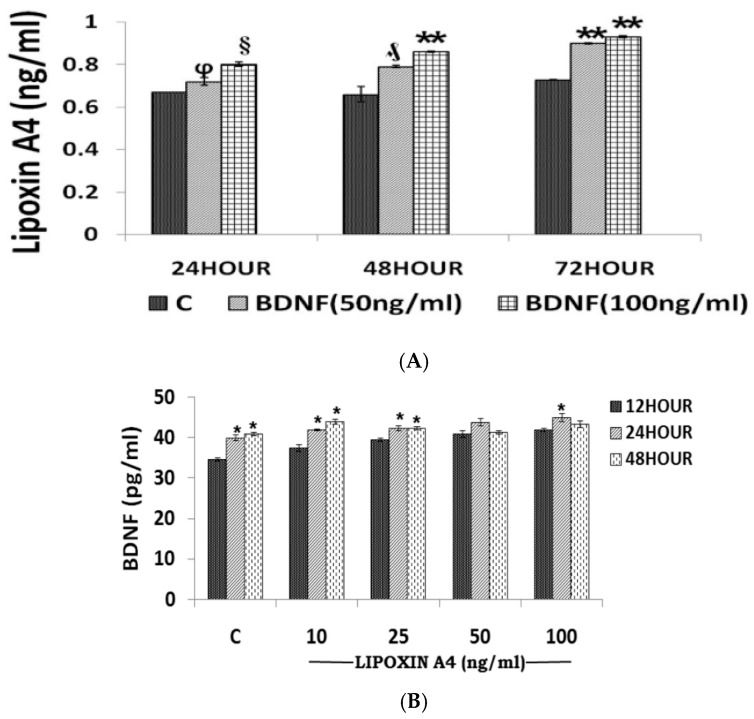
(**A**) Effect of BDNF (50 and 100 ng/mL) on the secretion of LXA4 by RIN5F cells in vitro after 24, 48, and 72 h of incubation. All values expressed as mean ± SEM. φ *p* < 0.001, § *p* < 0.05, ₰ *p* < 0.01, ** *p* < 0.001 vs. untreated control. BDNF, brain-derived neurotrophic factor. (**B**) Effect of LXA4 (10, 25, 50, and 100 ng/mL) after 12, 24, and 48 h of supplementation on BDNF secretion by RIN5F cells in vitro. All values expressed as mean ± SEM. * *p* < 0.001 vs. respective untreated control. The data are from reference [60].

**Figure 6:**
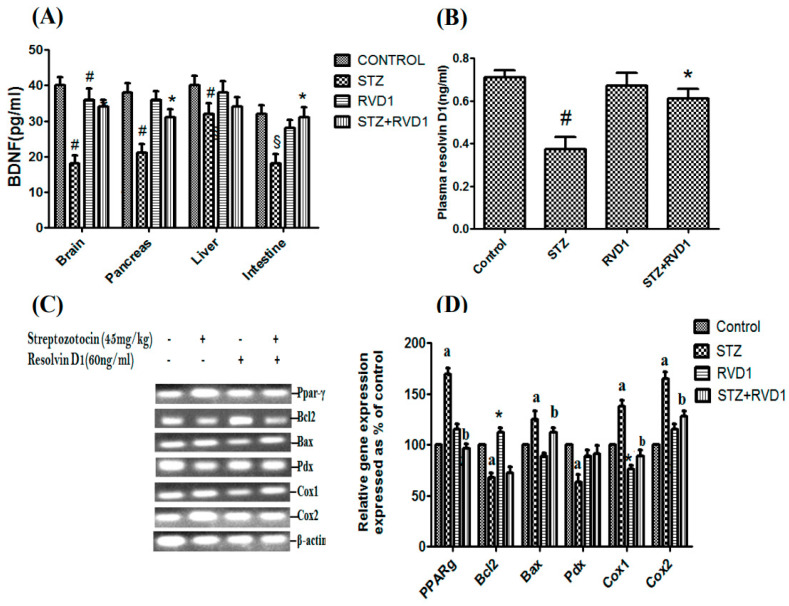
(**A**) Effect of RVD1 treatment on BDNF protein expression in the brain, pancreas, liver, and the intestine in T1DM and controls. Data are expressed as mean ± SEM. Brain: control vs. T1DM, # *p* ≤ 0.01, * *p* ≤ 0.05; pancreas: control vs. T1DM, § *p* ≤ 0.01; intestine: control vs. T1DM, # *p* ≤ 0.05. (**B**) Plasma RVD1 levels in various groups measured at the end of the study (day 30). * *p* ≤ 0.001 compared to untreated control and # *p* ≤ 0.05 compared to STZ (T1DM) control. (**C**,**D**) Effect of RVD1 (60 ng/kg) treatment on changes in the mRNA expression of PPAR-gamma, Bcl2, Bax, Pdx, Cox1, and Cox2 in pancreatic tissue. Pancreatic tissue was collected on day 30. The percentage of change in gene expression and β-actin was determined using the semi-quantitative PCR method. All values are expressed as mean ± SEM. (a) *p* ≤ 0.01, * *p* ≤ 0.05 compared to untreated control values and (b) *p* ≤ 0.001 compared to STZ (T1DM) control. These results suggest that RVD1 (resolvin D1), like LXA4, has potent anti-inflammatory properties that may explain its potential use in the prevention and management of DR. The data are from reference [60].

**Figure 7:**
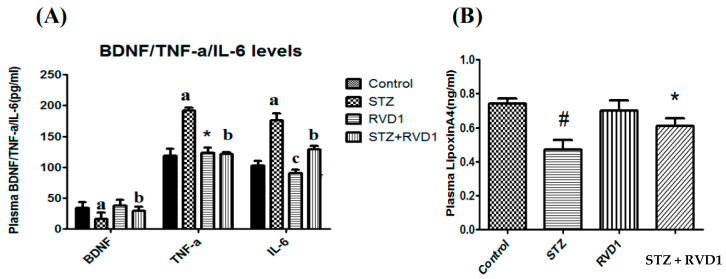
Effect of RVD1 treatment on plasma levels of BDNF/TNF-α/IL-6/LXA4. (**A**) Plasma BDNF/TNF-α/IL-6 levels. Plasma BDNF level in STZ + RVD1- vs. STZ (T1DM)-treated groups estimated at the end of the study (day 30). TNF-α studies: (a) *p* ≤ 0.001, * *p* ≤ 0.01 compared to control and compared to STZ control and (b) *p* ≤ 0.05 compared to STZ (T1DM); IL-6 studies: (a) *p* ≤ 0.01 and (c) *p* ≤ 0.05 compared to untreated control and STZ control values. (b) *p* ≤ 0.01 compared to STZ (T1DM) group. *p* ≤ 0.01. (**B**) Effect of RVD1 treatment on plasma LXA4 levels in STZ-induced type 1 DM animals. * *p* ≤ 0.01 compared to STZ (T1DM) and *p* ≤ 0.01 compared to untreated control;. All values are expressed as mean ± SEM. LXA4 levels in the plasma of STZ- and STZ + RVD1-treated groups measured at the end of the study (day 30). # *p* ≤ 0.001 compared to untreated control. * *p* ≤ 0.01 compared to STZ (T1DM) control (positive control group). The data are taken from reference [60]. Data shown in Figure 6 and Figure 7 are from reference [63].

It is evident from the preceding discussion that EFA metabolism is critical in the pathogenesis of DR. We previously reported that plasma phospholipid concentrations of AA, EPA, and DHA, the precursors of LXA4, resolvins, protectins, and maresins are decreased in those with type 2 diabetes mellitus and in experimental animals that were chemically induced to develop type 1 and type 2 diabetes mellitus [47,48,49,50,51]. Transgenic *fat-1* mice [52], which have high plasma and tissue concentrations of n-3 fatty acids and low AA levels, are resistant to chemically induced type 1 and high-fat diet-induced type 2 diabetes mellitus [53,54,55]. Alloxan-induced type 1 DM, streptozotocin-induced type 1 and type 2 DM, and high-fat diet-induced type 2 diabetes mellitus animals have low plasma and tissue (especially pancreas, liver, muscle, and adipose tissue) concentrations of GLA, AA, EPA, and DHA, suggesting a block or decrease in the activities of enzymes Δ^6^ and Δ^5^ desaturases ([49,50,51,52,56,57] see Table 5, Table 6, Table 7 and Table 8). This is supported by the observation that STZ-induced type 1 diabetic animals have decreased activities of desaturases in both the retina and liver; enhanced activity of COX-2, 5-LOX, and 12-LOX in the retina; and decreased plasma concentrations of GLA and AA, with no or little change in EPA and DHA [51]. In both type 1 and type 2 diabetes mellitus, increased plasma concentrations of PGE2, HETEs, and LTs (derived from AA) have been reported, indicating enhanced COX-2 and LOX activities [51,52,58,59]. Furthermore, both animal models of diabetes mellitus and patients with type 2 diabetes have low plasma and tissue concentrations of LXA4 (and, possibly, resolvins, protectins, and maresins) [46,51,52]. Paradoxically, *fat-1* mice induced to develop type 1 diabetes mellitus by streptozotocin showed high levels of pancreatic tissue LXA4 (derived from AA) and 18-HEPE (derived from EPA) compared to those in wild-type mice, whereas wild-type mice showed high levels of PGE2 and 12-HETE [53]. Both PGE2 and 12-HETE are pro-inflammatory, while 18-HEPE and LXA4 are anti-inflammatory in nature. These results suggest that despite the presence of enhanced amounts of EPA and DHA and low concentrations of AA in the pancreatic tissue, the resistance of *fat-1* mice to diabetes is due to the enhanced formation of LXA4 [53]. These results are in tune with our findings that AA and LXA4 prevent the development of both type 1 and type 2 diabetes [46,47,48,49,50,51,56,57,60]. LXA4 suppresses endothelial cell proliferation, free-radical generation, and VEGF and PDGF production but enhances the generation of BDNF, a cyto- and neuroprotective molecule [60,61,62]. Hence, it is likely that LXA4 can prevent DR (see Figure 5, Figure 6 and Figure 7).

**Table 5:** Fatty acid profile of the plasma phospholipid fraction of Wistar rats induced to develop type 1 diabetes by alloxan and the influence of insulin and Arasco oil (rich source of AA) on the same.

Percentage Distribution of Fatty Acids of Plasma Phospholipid Fraction of Wistar Rats Treated with Alloxan, Insulin, and Arasco Oil						
					Arasco Oil + Alloxan	
Fatty Acid	Control	Arasco Oil	Alloxan	Insulin	Pretreated	Simultaneously
	(n = 12)	(n = 12)	(n = 12)	(n = 12)	(n = 12)	Treated (n = 12)
16:0	23.15 ± 1.68	23.22 ± 0.94	23.64 ± 2.19	23.39 ± 2.48	22.20 ± 2.40	23.50 ± 1.61
18:0	17.20 ± 1.67	16.90 ± 1.72	16.63 ± 1.85	17.48 ± 2.38	11.80 ± 1.5 *†	12.9 ± 1.8 *†
18:1	11.21 ± 0.99	11.22 ± 1.08	12.39 ± 1.05 *	11.34 ± 1.03	11.28 ± 2.07	11.47 ± 1.36
18:2	21.64 ± 1.89	20.78 ± 1.71	22.88 ± 2.58	21.05 ± 1.97	24.29 ± 2.81 *	24.45 ± 3.78
18:3 w-6	0.50 ± 0.10	0.47 ± 0.13	0.26 ± 0.10 *	0.47 ± 0.11 †	0.32 ± 0.11 *	0.43 ± 0.18 †
20:3	0.89 ± 0.38	0.67 ± 0.17	0.57 ± 0.15 *	0.82 ± 0.33	1.39 ± 0.39 *†	0.47 ± 0.15 *
20:4	18.93 ± 2.46	21.44 ± 1.22 *	14.84 ± 1.35 *	19.1 ± 2.44	20.56 ± 3.63 †	19.6 ± 2.3 †
18:3/18:2	0.023	0.022	0.011	0.022	0.013	0.017
20:4/18:2	0.87	1.03	0.65	0.9	0.84	0.8
20:4/20:3	21.26	32	26.03	23.29	14.79	41.63
18:3 w-3	0.41 ± 0.17	0.33 ± 0.12	0.43 ± 0.21	0.43 ± 0.18	0.35 ± 0.16	0.32 ± 0.15
20:5	0.28 ± 0.09	0.50 +0.24 *	0.16 ± 0.09 *	0.24 ± 0.08	0.61 ± 0.39 *†	0.63 ± 0.3 *†
22:6	1.20 ± 0.32	1.18 ± 0.15	1.47 ± 0.32	1.28 ± 0.30	2.56 ± 0.27 *†	1.54 ± 0.28 *
20:5/18:3	0.68	1.51	0.37	0.55	1.74	1.96
22:6/20:5	4.28	2.36	9.18	5.33	4.19	2.44

* *p* ≤ 0.05 versus control group. † *p* ≤ 0.05 versus alloxan-treated group. The ratio between 18:3 and 18:2 ω-6 indicates Δ^6^ desaturase activity. The ratio between 20:4 and 18:2 ω-6 indicates the activities of Δ^6^ and Δ^5^ desaturases. The ratio between 20:4 and 20:3 ω-6 indicates the activity of the Δ^5^ desaturase enzyme. The ratio between 20:5 and 18:3 ω-3 indicates the activity of the enzyme Δ^6^ desaturase. The ratio between 22:6 and 18:3 ω-3 indicates the activities of the enzymes Δ^6^ and Δ^5^ desaturases. Alloxan-induced type 1 diabetic Wistar rats exhibited significantly reduced GLA (18:3 ω-6), GLA (20:3 ω-6), AA (20:4 ω-6), and EPA (20:5 ω-3) concentrations. These data also indicate that the activities of Δ^6^ and Δ^5^ desaturases were decreased (especially pertaining to the ω-6 fatty acids). The discrepancy in the concentrations of LA (18:2 ω6) and ALA (18:3 ω-3) metabolites indicates that there could be different isoenzymes of desaturases that act on LA and ALA. It is also seen that the decreased concentrations of AA, but not of GLA, DGLA, EPA, or DHA, could be restored to normal by AA-rich Arasco oil and insulin treatment. These data indicate that insulin activates desaturases.

**Table 6:** Fatty acid profile of the liver phospholipid fraction of Wistar rats induced to develop type 1 diabetes by alloxan and the influence of insulin and Arasco oil (rich source of AA) on the same.

Percentage Distribution of Fatty Acids of Liver Phospholipid Fraction of Wistar Rats Treated with Alloxan, Insulin, and Arasco Oil						
					Arasco Oil + Alloxan	
Fatty Acid	Control	Arasco oil	Alloxan	Insulin	Pretreated	Simultaneously
	(n = 12)	(n = 12)	(n = 12)	(n = 12)	(n = 12)	Treated (n = 12)
16:0	19.94 ± 2.74	18.99 ± 1.38	21.20 ± 2.87	20.68 ± 2.81	20.66 ± 2.71	20.18 ± 3.29
18:0	16.07 ± 2.22	17.83 ± 2.27	18.41 ± 2.23 *	17.2 ± 2.21	16.15 ± 1.94 †	16.76 ± 2.25
18:1	12.42 ± 1.49	10.28 ± 0.86 *	11.75 ± 1.35	11.78 ± 1.12	12.45 ± 2.38	12.25 ± 1.78
18:2	14.61 ± 2.05	12.10 ± 1.05 *	13.90 ± 2.14	14.01 ± 2.07	15.27 ± 2.46	14.91 ± 2.26
18:3 w-6	0.20 ± 0.04	0.18 ± 0.08	0.14 ± 0.03 *	0.18 ± 0.07	0.21 ± 0.07 †	0.21 ± 0.08 †
20:3	0.90 ± 0.28	0.75 ± 0.24 *	0.61 ± 0.17 *	0.82 ± 0.24	0.78 ± 0.19	0.89 ± 0.23 †
20:4	25.31 ± 3.66	31.87 ± 3.11 *	21.82 ± 2.36 *	24.43 ± 2.19 †	24.63 ± 2.78 †	24.78 ± 3.1 †
18:3/18:2	0.013	0.014	0.01	0.012	0.013	0.014
20:4/18:2	1.73	2.63	1.56	1.74	1.61	1.66
20:4/20:3	28.12	42.49	35.77	29.79	31.57	27.84
18:3 w-3	0.31 ± 0.09	0.31 ± 0.10	0.28 ± 0.10	0.30 ± 0.10	0.30 ± 0.09	0.28 ± 0.10
20:5	0.89 ± 0.30	0.95 ± 0.34	0.56 ± 0.14 *	0.81 ± 0.24 †	0.81 ± 0.22 †	0.87± 0.17 †
22:6	3.58 ± 0.63	3.01 ± 0.79 *	4.05 ± 0.72	3.84 ± 0.61	3.61 ± 0.58	3.74 ± 0.67
20:5/18:3	2.87	3.04	2	2.7	2.7	3.1
22:6/20:5	4.02	3.16	7.23	4.74	4.45	4.29

* *p* ≤ 0.05 versus control group. † *p* ≤ 0.05 versus alloxan-treated group. The ratio between 18:3 and 18:2 ω-6 indicates Δ^6^ desaturase activity. The ratio between 20:4 and 18:2 ω-6 indicates the activities of Δ^6^ and Δ^5^ desaturases. The ratio between 20:4 and 20:3 ω-6 indicates the activity of the Δ^5^ desaturase enzyme. The ratio between 20:5 and 18:3 ω-3 indicates the activity of the enzyme Δ^6^ desaturase. The ratio between 22:6 and 18:3 ω-3 indicates the activities of the enzymes Δ^6^ and Δ^5^ desaturases. Alloxan-induced type 1 diabetic Wistar rats exhibited significantly reduced GLA (18:3 ω-6), GLA (20:3 ω-6), AA (20:4 ω-6), and EPA (20:5 ω-3) concentrations. These data also indicate that the activities of Δ^6^ and Δ^5^ desaturases were decreased (especially pertaining to the ω-6 fatty acids). The discrepancy in the concentrations of LA (18:2 ω6) and ALA (18:3 ω-3) metabolites indicates that there could be different isoenzymes of desaturases that act on LA and ALA. It is also seen that the decreased concentrations of AA, but not of GLA, DGLA, EPA, or DHA, could be restored to normal by AA-rich Arasco oil and insulin treatment. The data indicate that insulin activates desaturases. Arasco oil is rich in AA (it contains 40% AA).

**Table 7:** Fatty acid profile of the skeletal muscle phospholipid fraction of Wistar rats induced to develop type 1 diabetes by alloxan and the influence of insulin and Arasco oil (rich source of AA) on the same.

Percentage Distribution of Fatty Acids of Muscle Phospholipid Fraction of Wistar Rats Treated with Alloxan, Insulin, and Arasco Oil						
					Arasco Oil + Alloxan	
Fatty Acid	Control	Arasco oil	Alloxan	Insulin	Pretreated	Simultaneously
	(n = 12)	(n = 12)	(n = 12)	(n = 12)	(n = 12)	Treated (n = 12)
16:0	22.83 ± 2.74	22.68 ± 1.75	23.53 ± 2.63	23.36 2.34	21.12 ± 2.50	22.13 ± 2.41
18:0	16.34 ± 1.66	15.23 ± 1.47	17.25 ± 1.47	17.10 1.65	15.78 ± 1.42	16.08 ± 1.55
18:1	9.57 ± 1.36	10.96 ± 1.47	9.38 ± 0.99	10.16 ± 1.86	9.93 ± 1.38	10.46 ± 1.55
18:2	20.03 ± 2.71	18.33 ± 1.31	20.77 ± 2.78	19.30 ± 2.39	20.61± 1.94	19.46 ± 2.22
18:3 w-6	0.44 ± 0.09	0.43 ± 0.08	0.24 ± 0.08 *	0.40 ± 0.09 †	0.44 ± 0.06 †	0.42 ± 0.08 †
20:3	0.43 ± 0.09	0.42 ± 0.11	0.31 ± 0.09 *	0.40 ± 0.10	0.42 ± 0.07 †	0.44 ± 0.07 †
20:4	20.42 ± 2.59	23.62 ± 2.32 *	17.23 ± 2.11 *	19.22 ± 2.14	22.15 ± 1.9 *†	21.71 ± 1.9 †
18:3/18:2	0.021	0.023	0.011	0.02	0.021	0.021
20:4/18:2	1.019	1.28	0.829	0.995	1.07	1.11
20:4/20:3	47.51	56.23	55.58	48.05	52.73	49.34
18:3 w-3	0.52 ± 0.12	0.49 ± 0.12	0.46 ± 0.10	0.51 ± 0.11	0.53 ± 0.11	0.52 ± 0.06
20:5	0.45 ± 0.09	0.51 ± 0.09	0.26 ± 0.07 *	0.43 ± 0.06 †	0.44 ± 0.10 †	0.44 ± 0.07 †
22:6	3.94 ± 0.78	3.79 ± 0.69	4.44 ± 0.74	4.11 ± 0.74	4.28 ± 0.60	4.43 ± 0.52
20:5/18:3	0.86	1.04	0.56	0.84	0.83	0.84
22:6/20:5	8.75	7.43	17.07	9.55	9.72	10.06

* *p* ≤ 0.05 versus control group. † *p* ≤ 0.05 versus alloxan-treated group. The ratio between 18:3 and 18:2 ω-6 indicates Δ^6^ desaturase activity. The ratio between 20:4 and 18:2 ω-6 indicates the activities of Δ^6^ and Δ^5^ desaturases. The ratio between 20:4 and 20:3 ω-6 indicates the activity of the Δ^5^ desaturase enzyme. The ratio between 20:5 and 18:3 ω-3 indicates the activity of the enzyme Δ^6^ desaturase. The ratio between 22:6 and 18:3 ω-3 indicates the activities of the enzymes Δ^6^ and Δ^5^ desaturases. Alloxan-induced type 1 diabetic Wistar rats exhibited significantly reduced GLA (18:3 ω-6), GLA (20:3 ω-6), AA (20:4 ω-6), and EPA (20:5 ω-3) concentrations. These data also indicate that the activities of Δ^6^ and Δ^5^ desaturases were decreased (especially pertaining to the ω-6 fatty acids). The discrepancy in the concentrations of LA (18:2 ω6) and ALA (18:3 ω-3) metabolites indicates that there could be different isoenzymes of desaturases that act on LA and ALA. It is also seen that the decreased concentrations of AA, but not of GLA, DGLA, EPA, or DHA, could be restored to normal by AA-rich Arasco oil and insulin treatment. The data indicate that insulin activates desaturases. Table 5, Table 6 and Table 7 are from [64].

**Table 8:** Fatty acid composition of the plasma phospholipid fraction in mice treated with STZ (streptozotocin)-induced type 1 DM. The data are from reference [51].

Fatty Acid Composition (%)	Group			
	N	STZ	STZ + ALA	STZ + LA
**SFA**				
Palmitic acid	22.63 ± 0.81 *	28.00 ± 1.24	23.70 ± 0.67 *	24.74 ± 0.30
(16:0)				
Stearic acid	16.55 ± 1.72	13.19 ± 1.16	16.72 ± 0.83	16.21 ± 0.69
(18:0)				
**MUFA**				
Palmitoleic acid	0.47 ± 0.07	0.27 ± 0.05	0.12 ± 0.04	0.17 ± 0.04
(16:1n7)				
Oleic acid	1.56 ± 0.15 *	0.89 ± 0.09	0.69 ± 0.05	0.75 ± 0.04
(18:1n9)				
**n6 PUFA**				
Linoleic acid	27.43 ± 2.70 *	14.05 ± 4.22	21.71 ± 1.73	26.93 ± 0.52 *
(18:2n6)				
γ linolenic acid	0.29 ± 0.06	0.16 ± 0.01	0.16 ± 0.01	0.16 ± 0.02
(18:3n6)				
Arachidonic acid	17.23 ± 1.71 *	11.75 ± 1.08	14.77 ± 1.85	15.95 ± 1.00 *
(20:4n6)				
**n3 PUFA**				
α linolenic acid	0.26 ± 0.01	0.18 ± 0.03	0.37 ± 0.07 *	0.29 ± 0.09
(18:3n3)				
EPA (20:5n3)	0.41 ± 0.03	0.40 ± 0.04	0.40 ± 0.01	0.41 ± 0.08
DHA (22:6n3)	2.57 ± 0.20	2.48 ± 0.34	5.64 ± 0.27 **	2.89 ± 0.17

**Figure 8:**
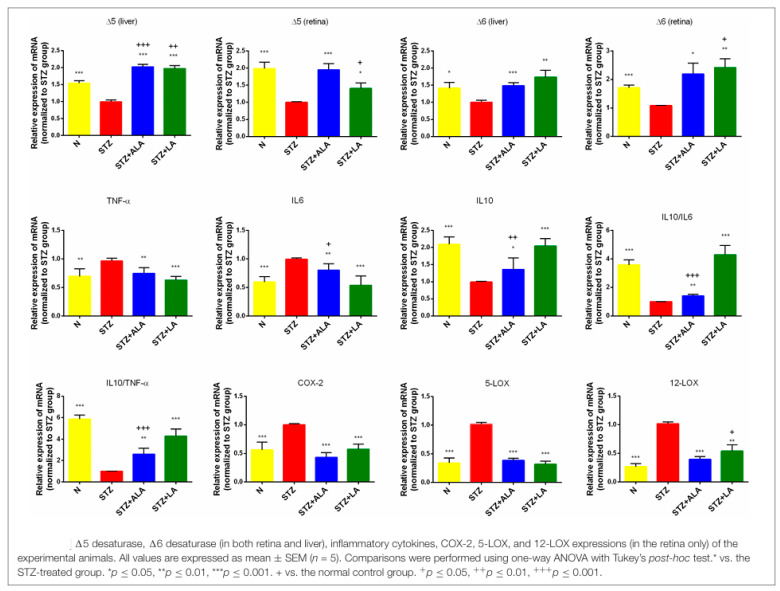
Relative expression of mRNAs of desaturases (in the retina and liver), COX and LOX enzymes, and pro-inflammatory (IL-6 and TNF-α) and anti-inflammatory (IL-10) cytokines (in the retina only); the ratio between pro- and anti-inflammatory cytokines in mice treated with STZ-induced type 1 DM; and the effect of supplementation of LA and ALA. It is seen that the desaturases are decreased, whereas COX and LOX activities are increased in the retina of STZ-treated animals. Both IL-6 and TNF-α are increased, while IL-10 is decreased in the retina of STZ-treated animals. These results suggest that in type 1 DM, the retina is inflamed, and the formation of LA and ALA metabolites (GLA, DGLA, and AA) occurs. The data are from [51].

Data are expressed as mean ± SEM (n = 5). SFA, saturated fatty acid; MUFA, monounsaturated fatty acid; PUFA, polyunsaturated fatty acid; EPA, eicosapentaenoic acid; DHA, docosahexaenoic acid. vs. STZ-treated group, * *p* < 0.05, ** *p* < 0.01. Plasma fatty acid composition of the phospholipid fraction in STZ-induced type 1 DM animals. The plasma concentrations of LA, GLA, and AA are low in STZ-induced type 1 DM and could be restored to near normal by LA supplementation. There were no changes in EPA and DHA concentrations, but ALA was low; this could be restored to normal by LA and ALA supplementation. These results indicate that ω-6 fatty acids have a more significant role in diabetes and the retina (DR) (see Figure 8). The data shown in the table are from Reference [51].

### 1.5. AA, EPA, and DHA and Their Metabolites in DR

Epidemiologic data indicated that EPA, DHA, and AA may prevent neovascular age-related macular degeneration and regulate retinal vaso-obliteration and neovascularization [65]. It was reported [66,67] that n-3-PUFA (EPA and DHA)-derived neuroprotectin D1, resolvin D1, and resolvin E1 protect against neovascularization by suppressing TNF-α [67]. We observed that both plasma and vitreous LXA4 and BDNF levels are low in patients with DR compared to those in controls (see Table 1, Table 2, Table 3 and Table 4) [46,56,57,60,68], and both LXA4 and BDNF have exhibit anti-diabetic actions and interact to potentiate each other’s actions and synthesis [69,70,71] (see Figure 5). Thus, there are critical roles for PGE2, 12-HETE, 18-HEPE, LXA4, resolvins, protectins, maresins, BDNF, cytokines, and VEGF in DR.

Anti-TNF-α therapy currently employed for DR is in line with the fact that plasma and vitreous VEGF levels are increased in DR [72,73,74,75]. LXA4 suppresses VEGF production in addition to its abilities to suppress NF-κB expression and function as a potent anti-inflammatory molecule [46,56,57]. This suggests that LXA4 may be better suited for the treatment of DR [76,77,78,79] (see Figure 5, Figure 6 and Figure 7). Similar protective action may also be seen with resolvins, protectins, and maresins, since they can enhance LXA4 production (see Figure 5, Figure 6 and Figure 7) [63,80].

In this context, the recent reports that there is a role for 12-LOX and its product 12-HETE, a pro-inflammatory molecule, in the pathogenesis of DR are interesting [81,82,83]. It is likely that 12-HETE can be converted to LXA4, though this has not been reported so far. Since there is normally a balance struck between pro- and anti-inflammatory compounds to maintain homeostasis, it is expected that such a balance exists between 12-HETE and LXA4 (see Figure 2) to maintain the integrity of the retina and its function. LXA4 exerts a negative control on PGE2, LTs, TXs, IL-6, TNF-α, and MIF (macrophage migration inhibitory factor) to suppress inappropriate inflammation. In a similar fashion, it is suggested that LXA4 may have a negative regulatory effect on 12-HETE synthesis and function. Hence, it is suggested that a deficiency in LXA4 results in an increase in 12-HETE. This implies that the intravitreal infusion/injection of LXA4 suppresses 12-HETE production and action, thus benefiting DR. This implies that the activities of PLA2 (phospholipase A2), 12-LOX, and 15-LOX are critical in the pathobiology of DR, since they are involved in the release of AA from the membrane lipid pool and the subsequent formation of 12-HETE and LXA4 (see Figure 2 and Figure 3).

### 1.6. Runt-Related Transcription Factor (RUNX1) in DR and Its Modulation by Bioactive Lipids (BALs)

Runt-related transcription factor (RUNX1) is expressed in human vascular endothelial cells [84]. In DR, RUNX1 expression is increased, whereas the inhibition of RUNX1 reduces vascular endothelial cell proliferation [85], implying that RUNX1 inhibitors may inhibit the progression of DR.

RUNX1 exhibits anti-inflammatory properties, and its deletion activates NF-κB [86]. 12-lipoxygenase (12-LOX, gene ALOX12), which catalyzes 12-HETE production from AA, is a direct transcriptional target gene of RUNX1. The knockdown of RUNX1 decreases 12-LOX proteins and thus may block the progression of DR [51,52,58,59,87]. This, coupled with the observation that COX-2, 5-LOX, and 12/15-LOX are overexpressed in STZ-induced diabetic animals [51] and HETE is increased in diabetes mellitus [53] with a concomitant decrease in LXA4 in the vitreous fluid in DR, suggests that LXA4 can suppress RUNX1–12-HETE expression and action [88,89,90,91,92,93,94,95,96,97]. Similarly, LXA4 may decrease the ability of neutrophils to release elastase enzymes in diabetes mellitus and DR [88,89,90,91,92,93,94,95,96,97].

### 1.7. AA, DHA, LXA4, Resolvins, and Protectins Are Safe and Show Anti-Inflammatory Properties

If bioactive lipids are to be employed in the prevention and management of DR, it needs to be established that they are safe and show adequate anti-inflammatory action in humans. A single intravitreal injection of 50 µg/50 µL, 25 µg/50 µL, or 5 µg/50 µL of DHA to healthy rabbits showed no adverse events [98]. LXA4 mitigated astrocyte reactivity in mouse retinas and human brain astrocytes exposed to cytokines, suggesting that it exerts neuroprotective properties [99,100]. Reductions in retinal *ALOX15* expression enhance the progression of retinitis pigmentosa (RP). Reduced *ALOX15* expression corresponded with the reduced formation of lipoxins (LXs), resolvins (RvDs), and docosapentaenoic acid-derived resolvins (DPA-RvDs). The decreased retinal DPA-RvD2 levels correlated with retinal structural and functional decline and the upregulation of microglial inflammation, suggesting that LXs and resolvins ameliorate retinal inflammation and thus are of benefit in DR and RP [101,102].

Posterior uveitis-induced retinal inflammation caused by LPS is reduced by lipoxins by virtue of their ability to inhibit CXCL9 (MIG) and CXCL10 (IP10), which are ligands for the CXCR3 chemokine receptor [103]. These results lend support to the concept that the lipoxin–CXCR3 pathway promotes distinct anti-inflammatory and proresolution pathways.

The ability of VIP (vasoactive intestinal peptide) to reduce hyperglycemia-induced increases in TNF-α, VEGF, ALX/FPR2, and GPR32 in human retinal vascular endothelial cells can be attributed to its ability to enhance resolvin D1 formation [104,105], suggesting that anti-inflammatory bioactive lipids are crucial to restore normal homeostasis. In this context, it is noteworthy that resolvin D1 enhances the production of LXA4, BDNF that of LXA4, and vice versa [58,62,76,77]. LXA4 is unlikely to exhibit significant toxicity, as supported by the observation that it reduced alkali-induced corneal inflammation and neovascularization and augmented the tissue repair process without any side effects [106].

### 1.8. Hyperglycemia-Induced Alterations in EFA Metabolism

Despite the preceding discussion about the potential role of EFAs and their metabolites in the pathobiology of DR, one major question that needs to be addressed is how and why hyperglycemia alters EFA metabolism. To sustain the argument that EFAs and their metabolites play a role in DR, it is important to show that hyperglycemia or factors that play a role in the induction of hyperglycemia and hyperglycemia-induced pathology are influenced by EFAs and their metabolites. For instance, it is known that hyperglycemic diabetics are more susceptible to infections, and these infections may be of a serious nature. This is in part because diabetic polymorphonuclear leukocytes (PMNs) show defects in several antimicrobial functions at the cellular level [107]. In this context, it is noteworthy that various metabolites of AA and other PUFAs play a regulatory role in PMN functions. This implies that the synthesis of various mediators of PMN function from AA are likely to be defective in diabetes. This leads to the suggestion that hyperglycemia may produce a deficiency of AA.

Animal studies have revealed that insulin is necessary for the normal physiological action of desaturases. Dietary linoleic acid (LA, 18:2 n-6) and alpha-linolenic acid (ALA, 18:3 n-3) are desaturated and elongated to their respective long-chain metabolites by Δ^6^ and Δ^5^ desaturases (see Figure 1). In situations wherein insulin deficiency occurs (as seen in type 1 DM), insulin action is defective, or insulin resistance is present, as seen in type 2 DM, the activities of desaturases will be defective [108]. Subsequent studies have revealed that insulin is more important than glucose per se for the activities of desaturases [109]; thus, it is insulin deficiency or resistance to insulin action is more critical than plasma glucose concentrations in the regulation of the activities of desaturases. In contrast to this, glucagon and epinephrine, which can enhance plasma glucose levels, depress the activity of Δ^6^ desaturase by increasing cAMP concentrations.

It is likely that low levels (which could be physiological) of lipid peroxides or their derivatives are required for several cellular functions, while excess lipid peroxides may be detrimental to cellular function and play a role in the development or occurrence of complications seen in diabetes. For instance, excess peroxides may be toxic to pancreatic islet beta cells, PMNs, and vascular endothelial cells (which may also show glutathione peroxidase deficiency). Thus, it is suggested that the accumulation of lipid peroxides impairs insulin secretion (whereas insulin suppresses lipid peroxide accumulation), inducing defects in PMN function and vascular endothelial cell dysfunction (this could include retinal, endothelial, and renal blood vessels). Thus, it is likely that insulin deficiency states and/or insulin resistance induce AA deficiency by decreasing the activities of desaturases that are needed for the conversion of dietary LA to AA. It is noteworthy that AA deficiency leads to the accumulation of excess lipid peroxides that are toxic to cells. This is supported by the observation that both alloxan- and streptozotocin-induced type 1 and type 2 DM animals have low AA content in their plasma and tissue (hepatic and muscle) phospholipid fractions [47,48,49,50,51] (see Table 5, Table 6 and Table 7 and Figure 8). It is paradoxical that normal/physiological concentrations of AA suppress the accumulation of lipid peroxides, whereas a deficiency of AA (and possibly other PUFAs) enhances the formation of excess of lipid peroxides [110] and that this, to some extent, is influenced by the GPX4 content of the cells. Under normal physiological conditions, a balance is maintained between the cellular content of PUFAs (especially AA) and GPX4. When the cell content of AA is decreased, there will be a decrease in GPX4 as well. When this decrease in AA content reaches a critical point, the decrease in GPX4 will be more dramatic, resulting in excess lipid peroxide formation, especially in cells that are exposed to mutagens, carcinogens, and diabetogenic agents (such as alloxan, STZ, and a high-fat diet), and this results in the excess formation of PGE2 and HETEs. In contrast, when the AA content in the cell is normal, the GPX4 content/expression will be normal, such that AA peroxidation will not occur, and the metabolism of AA will be directed more toward the formation of LXA4. This may be the reason as to why in AA deficiency states (such as DM), there is an enhanced formation of PGE2 and decreased generation of LXA4. In such instances, AA supplementation induces the generation of LXA4, decreases PGE2 and HETE formation, and triggers the adequate formation of GPX4 to prevent inappropriate lipid peroxidation processes.

The observation that both alloxan- and STZ-induced type 1 DM experimental animals [49,50,51] and patients with type 2 DM (with DR) have low plasma and retinal LXA4 levels [46], in conjunction with the fact that in DM (both type 1 and type 2), plasma and tissue concentrations of AA are decreased (Table 5, Table 6, Table 7, Table 8 and Table 9), suggests that precursor (AA) deficiency leads to the reduced formation of LXA4. Since LXA4 is a potent anti-inflammatory, cytoprotective, platelet anti-aggregative, vasodilative, and anti-angiogenic molecule, it stands to reason that its deficiency could be an important factor in the pathobiology of DR [76,77,78,79]. The ability of LXA4 to suppress TNF-α and IL-6 production and antagonize the actions of VEGF is of particular interest in view of their involvement in DR [79,111,112,113]. Furthermore, both IL-6 and TNF-α can suppress the activities of desaturases, which, in turn, aggravates the AA deficiency state. This results in an exacerbation of inflammation/inflammatory events caused by the enhanced formation of IL-6 and TNF-α due to the absence of negative feedback control exerted by AA and LXA4.

The altered EFA metabolism induced by alloxan, STZ, a high-fat diet, dyslipidemia, hyperglycemia, and insulin deficiency/insulin resistance can have profound effects on the formation of various eicosanoids. These include reduced prostacyclin (PGI2) synthesis with a concomitant increase in PGE2, PGF2α, TXA4 (thromboxane A2), and leukotrienes (LTs) in diabetes, even in the absence of an alteration in plasma lipid levels and body weight [114,115,116,117,118]. In addition to a decrease in the activities of desaturases, there is also an increase in COX-2 activity and an alteration in LOX activities, which contribute to the changes in various eicosanoids seen in T2DM. The major abnormality is in the metabolism of AA. The decreased activities of desaturases result in a deficiency of AA both in the plasma and in various tissues ([49,50,51] see Figure 8 and Table 9). AA is necessary for insulin secretion but does not require AA metabolism to go through COX-2 and 5-/12-LOX pathways [56,57,119,120]. AA exerts its actions via GPR120-mediated signaling events [121,122]. But the products of COX-2 and LOX activities have been implicated in the cytokine-mediated damage of β-cells, and, hence, selective inhibitors of these enzymes would be expected to play a dual protective role in diabetes: they may minimize β-cell dysfunction while maintaining insulin secretion by enhancing endogenous AA levels.

The observation that the treatment of RECs (retinal vascular endothelial cells) with HETE enhances ROS generation and the expression of NOX2 and pVEGF-R2 and decreases pSHP1 expression (pSHP-1 downregulates various proliferation pathways and is considered a potential angiogenesis regulator) suggests that the supplementation of diabetic mice with baicalein, a 12/15-LOX inhibitor, led to significantly decreased retinal levels of HETE, ICAM-1, VCAM-1, IL-6, ROS generation, NOX2 expression, and pVEGF-R2 while restoring pSHP1 levels in diabetic retina, indicating the potential contribution of 12/15-LOX to DR [81,82,83,123]. Since vitreous and plasma levels of LXA4 are reduced in DR [46], this suggests that a balance needs to be maintained between HETEs and LXA4 to maintain the integrity of the retina (see Figure 2 and Figure 3).

## 2. Conclusions

It is evident from the preceding discussion that DR is an inflammatory condition. The human retina is rich in AA and DHA, which are needed for RPE functional integrity and to prevent retinal degeneration. By virtue of its ability to enhance PEDF, DHA serves as a cytoprotective molecule not only for retinal cells but also for pericytes and vascular endothelial cells and thus plays a significant role in maintaining the integrity of the blood–retinal barrier. It is likely that metabolic stress (such as DM) enhances the production of pro-inflammatory cytokines, which, in turn, augment the generation of PGs, LTs, and TXs to produce inflammation and damage in various structures of the retina, resulting in DR. In contrast, production of adequate amounts of lipoxins, resolvins, protectins, and maresins will prevent DR. Hence, maintaining the delicate balance between pro- and anti-inflammatory molecules (cytokines, eicosanoids, and adhesion molecules) is crucial to maintain both retinal structural integrity and function to prevent DR. Since DM is associated with a deficiency of DGLA, AA, and DHA and the deceased formation of lipoxins, resolvins, protectins, and maresins with a concomitant increase in pro-inflammatory PGs (including HETEs), LTs, TXs, IL-6, TNF-α, and VEGF, it is suggested that restoring their balance to normal will prevent DR. It is proposed that the intravitreal injection of LXA4 may represent a new therapeutic approach to prevent and manage DR and other retinal conditions: AMD, macular edema, and RP. In addition, LXA4 is useful in other inflammatory conditions of the eye, such as keratitis and uveitis, and can enhance the healing of corneal ulcers. Hence, the potential use of the local application of LXA4 (resolvins, protectins, and maresins) for keratitis and corneal ulcers may be attempted, while intravitreal injections may be needed for DR, AMD, macular edema, RP, and uveitis. It is worth investigating whether the local instillation of LXA4 (resolvins, protectins, and maresins) as eye drops is suitable for DR, AMD, macular edema, and RP.

One needs to make a comparison to consider why LXA4 (resolvins, protectins, and maresins) is a better alternative to intravitreal anti-VEGF antibody and corticosteroids, which are currently employed in the treatment of DR. The effectiveness of anti-VEGF antibody and corticosteroids is not universal, and only 50–60% of the patients respond. It has also been documented that repeated injections of anti-VEGF antibody and corticosteroids are not without side effects.

The main current challenges and limitations in the use of anti-VEGF therapy for DR include limited anatomical and visual acuity improvements. Several studies reported that anti-VEGF therapy showed only moderate and limited anatomical improvements in visual acuity. It was reported that central retinal thickness decreased by −48 μm, and mean visual acuity increased by only +0.6 letters after 12 months of treatment. It was also noted that variable responses to anti-VEGF therapy could occur between different drugs. It is estimated that some patients need seven or more injections to show a reasonable response to anti-VEGF therapy. Some concerns have been raised regarding the potential renal effects of intravitreal anti-VEGF therapy. A retrospective cohort study found that some individuals with diabetic macular edema showed a worsening glomerular filtration rate (eGFR) and microalbuminuria after anti-VEGF therapy. Treatment interruptions could result in DR progression. Some patients treated with anti-VEGF antibody experienced complications such as vitreous hemorrhage, neovascular glaucoma, and traction retinal detachment, which resulted in a significant loss of visual acuity. Similarly, those who received intravitreal corticosteroids developed glaucoma, which resulted in a significant loss of vision. Furthermore, some patients may develop resistance to anti-VEGF and corticosteroid therapy [124,125,126,127]. These results suggest that more reliable and physiologically relevant therapies are needed for DR, and, hence, LXA4 could be a better alternative. LXA4 is a lipid and is non-antigenic, unlike anti-VEGF antibody. In contrast to corticosteroids and anti-VEGF antibody, LXA4 is unlikely to produce any systemic side effects or glaucoma. As it is a lipid, and the retina is rich in lipids, it is expected that LXA4 will easily be incorporated into retinal lipids with no side effects. LXA4 has a short half-life, rapid action, and is a naturally occurring endogenous molecule and hence is unlikely to have significant side effects.

It is seen that RVD1 enhances LXA4 levels, exerts potent anti-inflammatory action, and enhances BDNF levels like LXA4. 

It is evident from the results shown in Figure 5, Figure 6 and Figure 7 that there is a crosstalk among BDNF, LXA4, and resolvins (and possibly protectins and maresins). LXA4 and resolvin D1 suppress IL-6 and TNF-α and thus exert anti-inflammatory actions. LXA4 and resolvins enhance BDNF production, and BDNF, in turn, enhances LXA4 formation. Resolvin enhances LXA4 formation.

## Figures and Tables

**Table 1:** Serum cytokines in control, type 2 DM, and DR.

	Control (n = 26)	Diabetic (n = 25)	NPDR (n = 25)	PDR (n = 25)
IFN-γ (pg/mL)	3.21 ± 1.85	3.27 ± 2.19	5.31 ± 11.2	3.36 ± 1.66
TNF-α (pg/mL)	1.43 ± 0.85	1.47 ± 0.81	1.24 ± 0.51	1.73 ± 1.22
IL-10 (pg/mL)	1.67 ± 0.67	2.55 ± 1.78	1.93 ± 0.89	2.43 ± 1.03
IL-6 (pg/mL)	3.06 + 1.67	3.98 ± 3.06	4.86 ± 2.95 *↑	6.66 ± 4.78 *↑
IL-4 (pg/mL)	2.27 ± 1.15	1.82 ± 0.73	1.29 ± 0.56	1.92 ± 0.88
IL-2 (pg/mL)	1.82 ± 1.08	2.48 ± 1.37	1.64 ± 0.94	2.16 ± 1.63

Values are expressed as mean ± SD. * *p* < 0.05: control vs. respective group. ↑ Indicates increase value compared to macular hole.

**Table 2:** Cytokines in vitreous fluid. PDR, proliferative diabetic retinopathy.

Vitreous Parameter	Macular Hole	PDR
	(n = 20)	(n = 30)
IFN-y (pg/mL)	4.51 ± 2.39	4.92 + 1.48
TNF-α (pg/mL)	2.62 + 1.24	3.19 ± 0.9
IL-10 (pg/mL)	2.43 ± 1.0	3.33 ± 0.68 *↑
IL-6 (pg/mL)	21.34 + 35.95	291.09 + 537.08 *↑
IL-4 (pg/mL)	2.97 ± 1.34	2.82 ± 1.14
IL-2 (pg/mL)	2.08 ± 0.75	2.90 + 1.05

Values are means ± SD. * *p* < 0.05; comparison between respective group vs. control. ↑ Indicates increase value compared to macular hole.

**Table 3:** Serum VEGF, PEDF, BDNF, and LXA4 levels in control, type 2 DM, and DR.

	Control (n = 27)	Diabetic (n = 27)	NPDR (n=30)	PDR (n = 30)
BDNF (pg/mL)	73.45 ± 32.3	63.65 + 30.07	47.51 ± 25.37 *↓	45.86 ± 52.36 *↓
LXA4 (pg/mL)	127.95±108.2	84.54 ± 93.62	60.51 ± 51.70 *	50.27 ± 41.17 *
VEGF (pg/mL)	960.09 ± 876.6	660.41± 446.25	590.16 ± 422.26	960.09 ± 876.6
PEDF (μg/mL)	4.17 ± 2.17	4.97 ± 2.83	5.73 ± 2.57	5.76 + 3.34

Values are expressed as mean ± SD. * *p* < 0.05: control vs. respective group (Mann-Whitney U test). ↓ Indicates reduced value compared to macular hole.

**Table 4:** Vitreous levels of BDNF, VEGF, PEDF, and LXA4 in proliferative diabetic retinopathy and macular hole. Data shown in Table 1, Table 2, Table 3 and Table 4 are from reference [46].

Vitreous Parameter	Macular Hole	PDR
	(n = 18)	(n = 27)
BDNF (pg/mL)	50.44 ± 79.14	13.47 ± 28.56 *↓
LXA4 (pg/mL)	54.45 ± 40.45	25.63 ± 23.1 *
VEGF (pg/mL)	33.78 ± 29.24	971.75 ± 951.03 *
PEDF (μg/mL)	3.38 ± 3.66	7.98 ± 4.26 *
VEGF/PEDF ratio	85 ± 143.20	165+ 194.79

Values are expressed as mean ± SD. * *p* < 0.05 compared to macular hole. ↓ Indicates reduced value compared to macular hole.

**Table 9:** The percentage distribution of fatty acids in the plasma phospholipid fraction of patients with type 2 DM. All values are expressed as mean ± S.D. * *p* < 0.05 compared to control. These data are taken from reference [48].

Fatty Acid	Control (n = 20)	Type 2 DM (n = 10)
DGLA (20:3 n-6)	3.4 ± 1.0	1.7 ± 1.0 *
AA (20:4 n-6)	9.4 ± 1.8	4.6 ± 1.8 *
EPA (20:6 n-3)	0.4 ± 0.4	0.3 ± 0.3
DHA (22:6 n-3)	1.4 ± 0.5	0.5 ± 0.4 *
